# The Morals of Children

**Published:** 1897-08-21

**Authors:** 


### THE MORALS OF CHILDREN.

The *Gentlewoman* has discovered the existence of "a little paper called The Hospital," and has therein read the statement that children are innately thieves and liars. The statement horrifies her, but she valiantly overcomes her "contempt for the person" who makes it to the extent of giving him the lie direct. The *Gentlewoman* holds that children are honest and truthful until cowardice, born of reproof or punishment, makes them conceal their acts. The difference in opinion seems to the "contemptible person" to be largely a difference in the use of words. Children take anything that catches their eye, do anything that pleases them, without regard to prior rights. It is after these prior rights have been explained to them that The Hospital writer took up the case—as soon, that is, as consciousness can grasp the fact of responsibility. The *Gentlewoman* wants to start earlier—how early it would be difficult to say; for a three months old baby, who will break your Sèvres vase with a light heart, will protest most vigorously if you pretend to take away its rattle. It understands *meum* even thus early, but has not learned the importance of *tuum*. That must be taught it, and by the time a child can walk and talk it usually has clear enough ideas as to the distinction between its own and other people's property. Yet the average child, not necessarily "hereditarily depraved" as the *Gentlewoman* thinks—bless her innocent, lady-like heart!—will trespass among the strawberry beds, or ravel the wools in the work-basket, or touch the china in the drawing-room, even though it is conscious that the gardener, or nurse, or mother will be angry if they find out; and when they do find out, and are angry, the child's natural instinct is to deny responsibility, or, if older and more ingenious, to explain it away. George Washington's "I cannot tell a lie" would not have become historic if he had only done what nine out of ten children would naturally do when accused of a fault. If our straightforward words offend the *Gentlewoman* we will say "children naturally take anything they want, without regard to prior ownership, and do not admit responsibility for actions which they have learned are regarded by others as blameworthy"; but does not this mean just the same as "children are thieves and liars"? They learn to lie through cowardice, it is true, though it be only the noble cowardice of not wishing to see a mother's face look grieved; but this cowardice is a necessary step in their moral evolution, until they have grasped the fact that the things that grieve mother are things which are intrinsically wrong; this again leading to apprehension of the fact that these things grieve mother —and beyond mother, God—because they cause loss or injustice to others. And at this point conscience, which "doth make cowards of us all"—that is indeed its function—will show him that the greatest injustice we can do others is to let them bear the blame or punishment for our offences. Thence comes the courage to admit responsibility, even if we suffer by it; thence comes what may be accurately defined, and is popularly meant, by "telling the truth," as opposed to merely "stating a fact," which the *Gentlewoman* seems to regard as identical. The latter is a merely intellectual act; the former is intellectual *plus* moral. Whether it is to be deplored or not, it remains the rule of human experience that only by consciousness of sin, even when this is not acquired by personal sin, can we attain to higher righteousness. As we said in our article, the child's vices are unconscious, and, "before punishing them as moral defects, you must instruct the child in the fact that they are such." The *Gentlewoman* argues that because the acts are not consciously vicious they are not vicious at all. It is then our business, our duty, to awaken that consciousness, for the sake of our children and of the welfare of the community, of which we and they are members. We hope that after this explanation the *Gentlewoman* will return to her chiffons with an easy mind, convinced that The Hospital does not regard children as fiends incarnate, but only wished to point out a stage in the evolution of the individual which cannot but cause anxiety to parents. The African savages do not, according to the *Gentlewoman*, pass through that stage. But then, do we want our children to be savages?

